# Estimation of dihydroartemisinin in human plasma using a highly sensitive LTQ Orbitrap mass spectrometer with Xcalibur software

**DOI:** 10.3389/fphar.2023.1157604

**Published:** 2023-05-22

**Authors:** Tareq Abu-Izneid, Muhammad Abbas, David G. Watson, Yasar Shah, Sayyed I. Shah, Fazli Khuda

**Affiliations:** ^1^ Pharmaceutical Sciences, College of Pharmacy, Al Ain University, Al Ain, United Arab Emirates; ^2^ Department of Pharmacy, Abdul Wali Khan University Mardan, Mardan, Pakistan; ^3^ Strathclyde Institute of Pharmacy and Biomedical Sciences, University of Strathclyde, Glasgow, United Kingdom; ^4^ Department of Pharmacy, University of Peshawar, Peshawar, Pakistan

**Keywords:** antimalarial, artemether, metabolites, LC-MS, analytical method, dihydroartemisinin, human plasma

## Abstract

**Background:** Artemether (ARM), the O-methyl ether prodrug of dihydroartemisinin (DHA), is considered a first-line antimalarial agent. Artemether is extensively metabolized *in vivo* to its active metabolite DHA, and therefore its determination offers considerable difficulties. In the present study, DHA identification and estimation were accurately performed by the mass spectrometric analysis, using a high-resolution liquid chromatography/electrospray ionization-mass spectrometry (LC/ESI-MS) LTQ Orbitrap hybrid mass spectrometer.

**Methods:** The plasma samples were taken from healthy volunteers, and the spiked plasma was extracted by adding 1 mL of a mixture of dichloromethane and *tert*.-methyl butyl ether (8:2 v/v) to 0.5 mL of plasma. The internal standard solution (artemisinin 500 ng/mL) was added to the plasma samples. After vertexing and centrifugation, the organic layer was separated and transferred into another tube and dried under nitrogen. The residue was reconstituted in 100 μL of acetonitrile and was injected onto the LC-MS system for analysis. Measurement of standards and samples was carried out isocratically on a Surveyor HPLC system combined with an LTQ Orbitrap mass spectrometer using an ACE 5 C18-PFP column. Mobile phase A consisted of 0.1% v/v formic acid in water, Mobile phase B consisted of acetonitrile only, and isocratic elution was carried out with A:B 20:80, v/v. The flow rate was 500 μL/min. The ESI interface was operated in a positive ion mode with a spray voltage of 4.5 kV.

**Results:** Artemether is not a very biologically stable compound and is immediately metabolized to its active metabolite dihydroartemisinin, so no clear peak was observed for artemether. Both artemether and DHA after ionization undergo neutral losses of methanol and water, respectively, in the source of the mass spectrometer. The ions observed were (MH-H_2_O) m/z 267.15 for DHA and (MH-m/z 283.15 for internal standard artemisinin. The method was validated according to international guidelines.

**Discussion:** The validated method was applied successfully for the determination and quantification of DHA in plasma samples. This method works well for the extraction of drugs, and the Orbitrap system with the help of Xcalibur software accurately and precisely determines the concentration of DHA in spiked as well as volunteer’s plasma.

## 1 Introduction

Malaria causes widespread morbidity and mortality in the developing countries and hence a major cause of public health problems for more than a century. According to the World Malaria Report 2022, approximately 247 million malaria cases were reported throughout the world in 2021, with approximately 625,000 estimated deaths reported across the world in 2020 (Organization, 2022), while in 2018, 228 million cases and 405,000 deaths were reported (Organization, 2020), which imparts huge financial burden and impaired social and economic growth of the affected regions. Although the disease is confined to inter-tropical regions, the population living at risk counts for almost three billion with exponential growth (Guinovart et al., 2006). In addition to the failure to eradicate larvae, the outcomes of the disease have become more serious due to the occurrence of resistance by malarial parasites to the available antimalarial drugs like chloroquine, sulfadoxine, pyrimethamine, and mefloquine. However, there is a continuous struggle for the development of new antimalarial entities, combination of existing drugs, active targeting, and candidate vaccines to combat the disease. Artemisinin was reported as a potent drug extracted and purified from *Artemisia annua* (Klayman, 1985), the leafy herb used for centuries in China for treating fever and malaria. Artemisinin and its several derivatives showed promising results in declining the mortality rate of malaria since they are commercially available for clinical use (Edikpo and Adikwu, 2013).

**FIGURE 1:**
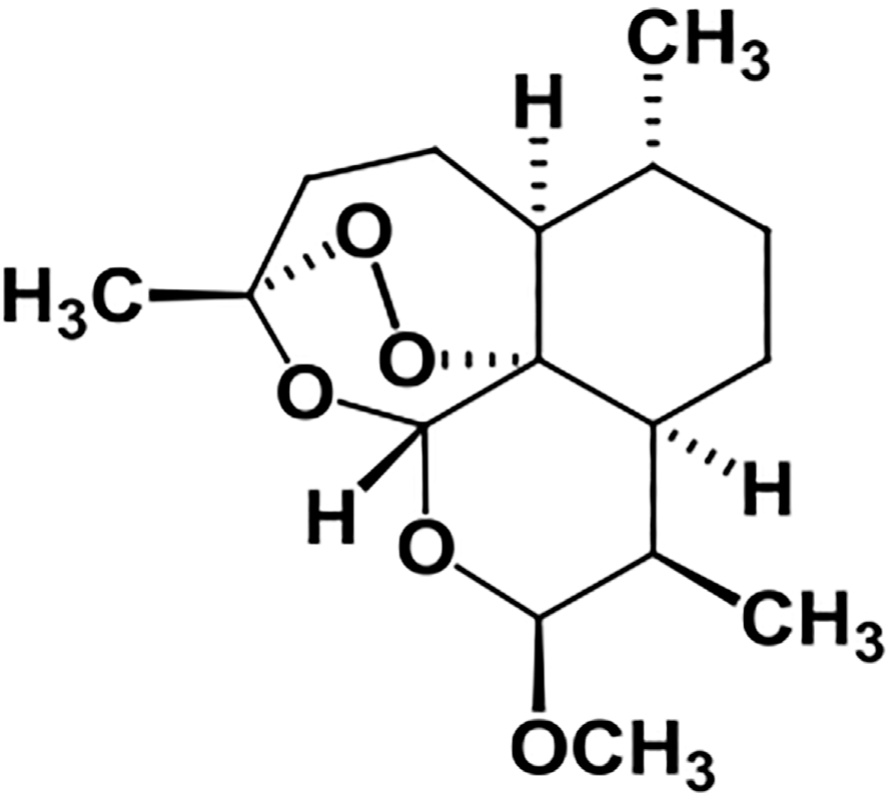
Structure of artemether.

Artemether (Figure 1) is a lipophilic drug and a methyl ether derivative of artemisinin (Liu et al., 2011; Raju and Taneja, 2013), having a logP value of 3.5 (Matsa et al., 2019).

**FIGURE 2:**
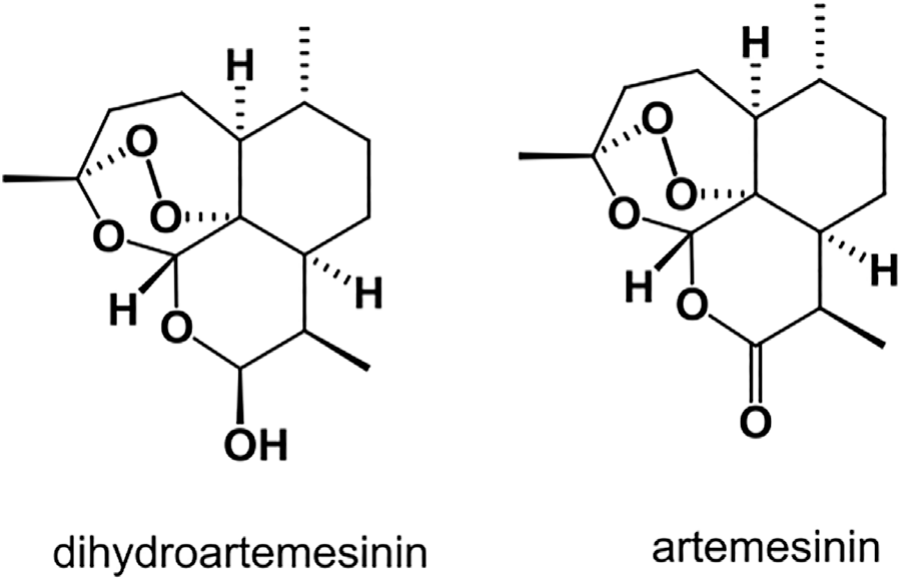
Structure of dihydroartemisinin (DHA) and artemisinin.

Chemically, artemisinin (Figure 2) and its derivatives are sesquiterpene trioxane lactones and contains a peroxide bridge which is necessary for its antimalarial activity. The reduction of the lactone with sodium borohydride produces dihydroartemisinin (Figure 2) which also possesses high antimalarial activity (Peys et al., 2005; Pinheiro et al., 2018).

Artemether confers high instability inside the human body and rapidly metabolizes to an active metabolite dihydroartemisinin (DHA). Determining artemether in the human plasma is difficult and misleading due to its rapid conversion to DHA, despite both molecules contributing to antimalarial efficacy. Therefore, quantification of DHA in human plasma is a good alternative to demonstrate the concentration of artemether in systemic circulation. A number of analytical methods have been reported for the quantification of artemether and its metabolite DHA (Hodel et al., 2009; Huang et al., 2009; Yang et al., 2018). These studies mainly involve determination of artemether only using the tandem mass spectrometer. The other techniques used for the determination of antimalarials include thin-layer chromatography, gas chromatography, spectroscopic, and immunological methods. The most commonly used methods are based on high-performance liquid chromatography coupled with either ultraviolet detection, evaporative light scattering detection, electron capture detection, or electrospray ionization and mass spectrometer detection (Vandercruyssen et al., 2014). However, these techniques are associated with expensive extraction procedures and less sensitivity. This study involved the use of high-resolution liquid chromatography/mass spectrometry (LC-HR/MS) experiments in a LTQ Orbitrap mass spectrometer that provided robust accurate mass data for unambiguous determinations of elemental compositions. In the present study, the structural characterization and identification of the metabolite of artemether were achieved by data-dependent accurate mass spectrometric analysis using an LC-HR-MS/MS system composed of an LTQ Orbitrap mass spectrometer. Its sensitivity and resolution are better than those of the other technologies using mass spectrometry systems. The method accurately determines the mass of the target compound and performs the MS/MS of the target compound. Xcalibur software was used as it is simple and also provides the exact mass formula and chemical composition of the sample (Zubarev and Makarov, 2013).

## 2 Experimental

### 2.1 Chemicals and solvents

Artemether (purity ≥99%) C_16_H_26_O_5_ Mol. Wt 298.38, DHA C_15_H_24_O_5_ Mol. Wt 284.35, internal standard artemisinin (purity ≥98%) C_15_H_22_O_5_ Mol. Wt 282.33, dichloromethane, and *tert*.-ethyl butyl ether were obtained from Sigma-Aldrich. HPLC-grade acetonitrile (ACN) was purchased from Fisher Scientific, United Kingdom HPLC-grade water was produced by a Direct-Q 3 Ultrapure Water System from Millipore, United Kingdom. AnalaR-grade formic acid (98%) was obtained from BDH-Merck, United Kingdom. Drug-free human plasma was provided by Golden Jubilee Hospital, Glasgow, and was stored at −80°C.

### 2.2 Liquid chromatography-mass spectrometry analysis

Measurement of standards and samples was carried out isocratically on a Surveyor HPLC system combined with an LTQ Orbitrap mass spectrometer (Thermo Fisher Scientific, Hemel Hempstead, United Kingdom). An ACE 5 C18-PFP column (150 × 4.6 mm, 5 μm, Hichrom, Reading, United Kingdom) fitted with a guard column having the same packing material was used. Mobile phase A consisted of 0.1% v/v formic acid in water, Mobile phase B consisted of acetonitrile only, and isocratic elution was carried out with A:B 20:80, v/v. The flow rate was 500 μL/min. The ESI interface was operated in a positive ion mode with a spray voltage of 4.5 kV. The temperature of the ion transfer capillary was 275°C, and the flow rates of the sheath and auxiliary gases were 50 and 17 arbitrary units, respectively. The full scan range was m/z 75–1,200. The data were recorded using Xcalibur 2.1.0 software (Thermo Fisher Scientific). Mass calibration was performed for both ESI polarities before the analysis using the standard Thermo Calmix solution, and the signals at 83.0604 m/z (2xACN + H) were selected as lock masses for the positive ion mode during each analytical run.

### 2.3 Preparation of standards

Primary stock solutions for artemether, DHA, and internal standard artemisinin were prepared by dissolving 1 mg in 1 mL of acetonitrile. Working solutions of variable concentrations were prepared from the stock solution in acetonitrile. The working solution was spiked into blank plasma to obtain calibration samples of different concentrations (Section 3.4). All the stock and working solutions of DHA and IS were stored at 4°C, and all the spiked human plasma samples were stored at −80°C in a freezer between uses.

### 2.4 Preparation of plasma samples from human volunteers and spiked plasma

The plasma samples taken from the volunteers were thoroughly vertexed and then pipetted out. A volume of 500 μL solution was transferred into Eppendorf tubes. The internal standard solution (artemisinin 500 ng/mL) was added to the plasma samples. Dichloromethane and *tert.*-methyl butyl ether (8:2 v/v) were added as an extraction solvent, and the mixture was then vortexed for 5 min at 10,000 rpm. The organic layer was separated and transferred into another tube and dried under nitrogen. The residue was reconstituted in 100 μL of acetonitrile and was injected onto the LC-MS system for analysis.

### 2.5 Validation of the method

#### 2.5.1 Selectivity

The selectivity of the method was evaluated by assaying six different lots of analyte-free human plasma. These samples were compared to those containing artemether or dihydroartemisinin at the LOQ or artemisinin at 500 ng/mL.

#### 2.5.2 Accuracy and precision

The precision and accuracy of the method were determined by repeated preparation (x 5) of the 0.5 and 1.0 μg/mL spiked samples of plasma. These samples were processed in the same way as the plasma samples from volunteers.

#### 2.5.3 Calibration

Diluted stock solutions of dihydroartemisinin (DHA) were prepared at a concentration of 10 μg/mL and stored at 4°C, and a diluted stock solution of the artemisinin internal standard was prepared at 0.5 μg/mL. Then, 1 mL aliquots of plasma were spiked with 0 μg, 0.01 µg, 0.03 µg, 0.05 µg, 0.1 µg, 0.2 µg, 0.5 µg, 1 μg, 1.5 µg, 2 µg of DHA, and 0.5 μg/mL of artemisinin as an internal standard. Furthermore, these samples were processed in the same way as volunteer’s plasma samples. A linear range was thus established in the range of 0.01–2.0 μg/mL.

#### 2.5.4 Recovery, ion suppression, and robustness

The recovery from the protein precipitation procedure was determined by treating six replicates of plasma without the internal standard. The internal standard was then added to the extracts from plasma in the reconstitution step.

An ionization suppression test was carried out by mixing 0.4 mL of blank plasma with 3.2 mL of acetonitrile, and the sample was centrifuged and the supernatant was removed. The supernatant (500 µL) was spiked with 50 µL of DHA (0.5 μg/mL); similarly, 500 µL of acetonitrile was also spiked with 50 µL of DHA (0.5 μg/mL). The samples were then analyzed.

An aliquot of plasma sample (1 mL) was spiked with DHA to produce a concentration of DHA at 1 μg/mL above the original concentration to check the robustness of the method.

#### 2.5.5 Sensitivity

The sensitivity of the method was determined in standard solutions and blank plasma spiked with the analyte. The limit of detection was defined as the minimum concentration at which a signal-to-noise ratio of 3 was obtained, and for LOQ, a signal-to-noise ratio of 10 was considered.

#### 2.5.6 Application of the method to plasma samples

The method was further assessed by measuring dihydroartemisinin (DHA) concentrations in plasma samples collected from 26 healthy human volunteers. The study was conducted according to the guidelines of “World Medical Association’s Declaration of Helsinki—Ethical Principles for Medical Research Involving Human Subjects” and was approved by the Ethics Committee of the Department of Pharmacy, University of Peshawar. The samples were taken from healthy volunteers, and after processing, the samples were sent to the University of Strathclyde, Glasgow, United Kingdom, for LC-MS analysis. The participants were asked to follow the standard protocol by not taking any medication for at least 7 days prior to and during the clinical trial. In a double-blind study, the participants were divided into two groups. In the first phase, one group was given pomegranate juice for 7 days and then Coartem^®^ tablet was given orally, while Coartem^®^ tablet was given with water to the other group. In the second phase, the order was reversed. The blood samples were taken at different time intervals for the determination of DHA at predose and at 0.25, 0.5, 1, 1.5, 2, 2.5, 3, 4, 6, 8, 10, and 12 h after taking Coartem^®^. The samples were then processed immediately and stored at −80°C before being analyzed by LC-MS after reconstitution.

## 3 Results

### 3.1 LC-MS optimization

Different C-18 columns were tested for the efficient determination and detection of DHA in human plasma. Among the different columns tested, an ACE 5 C18-PFP column (150 × 4.6 mm, 5 μm, Hichrom, Reading, United Kingdom) fitted with a guard column having the same packing material was selected as the best column based on better peak resolution and shape.

**FIGURE 3:**
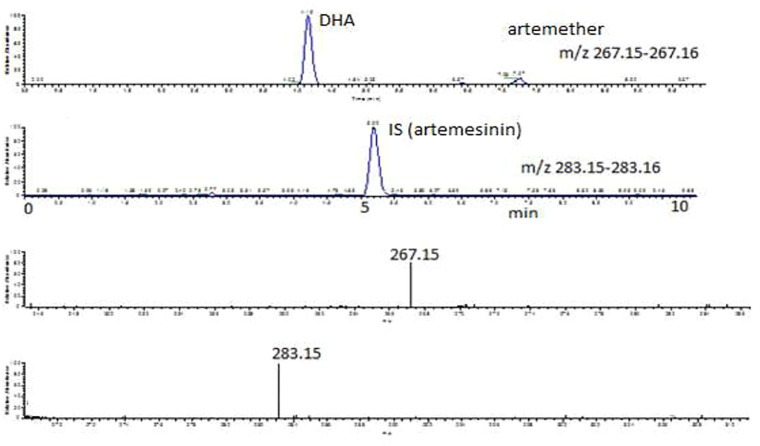
Extracted ion chromatograms and mass scan spectra of artemether, DHA (1 μg/mL), and IS (0.5 μg/mL) in a standard solution.

**FIGURE 4:**
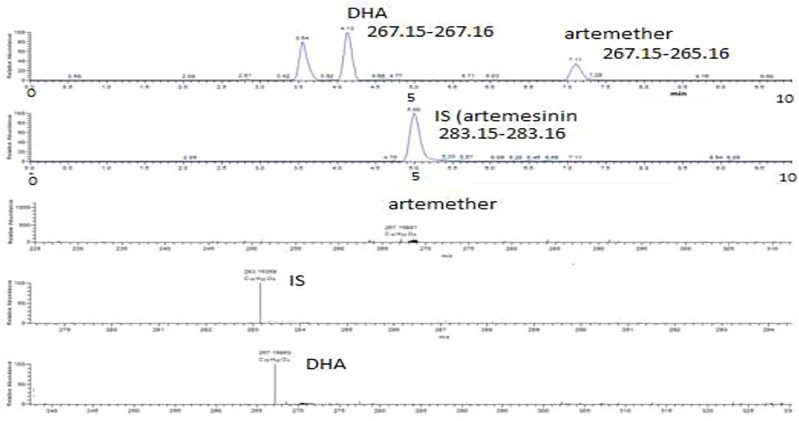
Extracted ion chromatograms and mass scan spectra of artemether, DHA, and IS extracted from plasma obtained from volunteers. The concentration of IS was 0.5 μg/mL.

**FIGURE 5:**
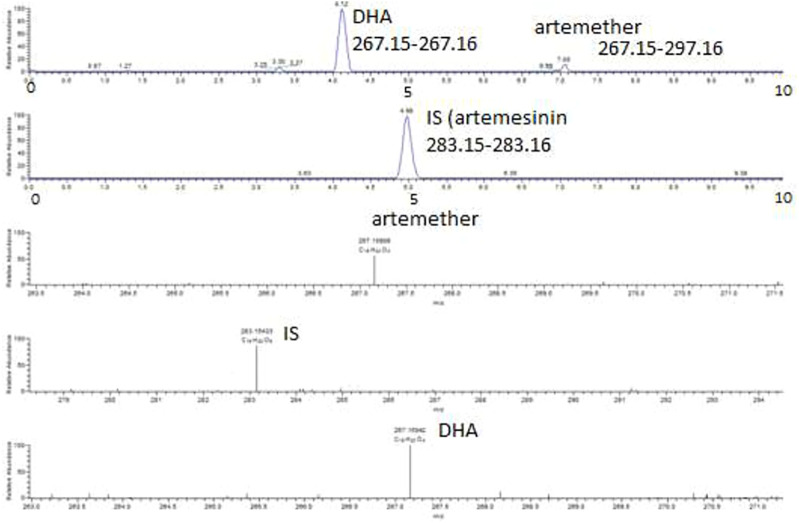
Extracted ion chromatograms and mass scan spectra of artemether, DHA, and IS in spiked plasma. The concentration of artemether in the spiked plasma was 1 μg/mL, while the concentration of IS artemisinin was 0.5 μg/mL.

For the determination of DHA in plasma, various parameters of the mobile phase were tried. Initially, ammonium acetate buffer was used in the mobile phase to promote formation of the ammonium adduct of artemether according to César et al. (2011), but there was no clear peak observed for the ammonium adduct of artemether or DHA. Then, 0.1% FA in acetonitrile and 0.1% FA in water were evaluated in different ratios, and the results were still not good. There was also tailing observed in the peaks detected for artemether, which may be due to the degradation of the compound at low pH. Then, 0.1% FA in water and acetonitrile was tried in different ratios, and the best results were obtained with 20:80 of 0.1% FA in H_2_O:ACN. The addition of formic acid to the mobile phase helps improve the peak shape and promotes the ionization of a compound in a positive mode. Thus, the method was reasonably robust although these analytes are difficult to be analyzed under ESI conditions since there is no particular center which can be readily protonated. The chromatographic and ESI mass spectra of artemether, DHA, and the internal standard artemisinin in a standard solution and in plasma samples at different concentrations are shown in Figures 3–5.

### 3.2 Selectivity

Artemether is extensively metabolized to its metabolite (DHA) in the liver which possesses a stronger antimalarial activity than the parent drug (Karbwang et al., 1997; Gallay et al., 2018). As artemether is not a very biologically stable compound and was immediately metabolized to its active metabolite DHA, no peak was observed for artemether. Therefore, DHA was considered the active metabolite of artemether and the main compound of interest to be quantified. Both artemether and DHA after ionization undergo neutral losses of methanol and water, respectively, in the source of the mass spectrometer, and these ions were used to quantify the compounds. The internal standard artemisinin was stable, and the protonated molecule was detected at m/z 283. The ions observed were as follows: (MH-CH_3_OH) m/z 267.15 for artemether, (MH-H_2_O) m/z 267.15 for DHA, and (MH-m/z 283.15 for internal standard artemisinin. The observed retention times were approximately 7.1 min for artemether, 4.1 min for DHA, and 5 min for the internal standard artemisinin.

### 3.3 Accuracy and precision

The values obtained for the accuracy and precision of the analysis of spiked plasma at 0.5 μg/mL and 1.0 μg/mL were 93.3% ± 2.7% and 95.2% ± 1.7%, respectively (*n* = 5). Then, two plasma samples from volunteers were selected and were repeatedly analyzed (*n* = 5). The results were well within limits. Thus, both the method and instrument work well for the accurate analysis of DHA in plasma samples. There were two peaks observed for the plasma samples obtained from volunteers which according to Pichini et al. (2003) are due to *α*- and *ß*-tautomers of DHA. The formula of DHA was C_15_H_23_O_4_ corresponding to the peak at m/z 267 after the neutral loss of an H_2_O molecule from DHA at 5 ppm. The formula of IS was C_15_H_23_O_5_ corresponding to the peak at m/z 283.

### 3.4 Calibration

**FIGURE 6:**
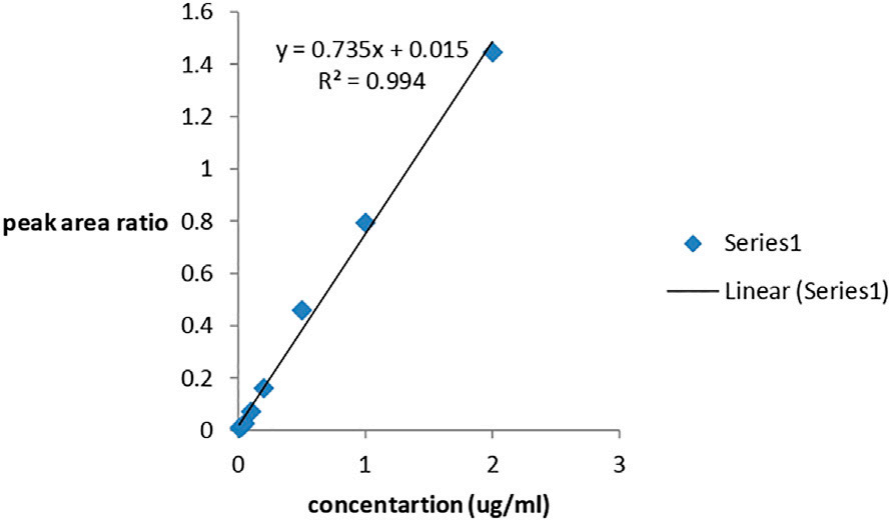
Calibration curve for DHA.

The ion at m/z 267 was the most suitable for the quantification of dihydroartemisinin (DHA), and a calibration curve was prepared. Solutions of varying amounts of DHA (for calibration, accuracy and precision, and stability assessments) and a fixed amount of the IS artemisinin were prepared in acetonitrile ranging from 0.01 μg/mL to 2 μg/mL and were injected to the HPLC-MS system. The concentration of DHA in micrograms was then plotted against the peak area ratio of DHA/IS, and the response was reasonably linear (R^2^ = 0.994). The equation of the calibration plot was y = 0.735x + 0.015. The calibration line is shown in Figure 6.

### 3.5 Recoveries, ion suppression, and robustness

In the recovery test, the recovery after liquid–liquid extraction with dichloromethane and *tert*.-methyl butyl ether (8:2 v/v) of 0.5 μg/mL spiking of DHA into plasma was 91.7% ± 8.9% (*n* = 6) and for 1.0 μg/mL spiking, the recovery was 90.1% ± 3.7% (*n* = 6).

In the ion suppression test, the ratio of the signal for 0.5 μg/mL spiking of DHA into the supernatant from a plasma crash compared with acetonitrile was 1.009% ± 5.4% (*n* = 2).

The robustness of the methodology was tested by spiking DHA into a patient sample already containing DHA to further assess ion suppression effects and recovery. In the sample selected, the initial levels of the drug were 0.389 μg/mL. The level of the drug after spiking at 1 μg/mL was 1.38 μg/mL ± 0.68% (*n* = 3).

### 3.6 Stability

The standard solutions were found to be stable for at least 1 month when stored at approximately −80°C. Therefore, the isolated plasma was stored at −80°C until further analysis.

### 3.7 LOD and LOQ

The LOD for dihydroartemisinin (DHA) was found to be 8 ng/mL in standard mixtures, while LOQ was found to be 20 ng/mL in spiked plasma samples.

### 3.8 Application of the method

**FIGURE 7:**
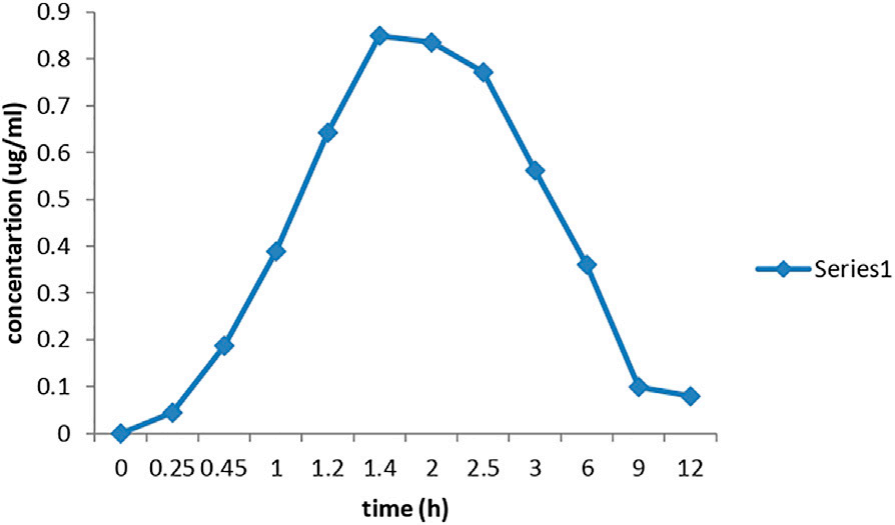
Concentration of DHA at different time intervals in the plasma obtained from volunteers.

The method was applied for determining DHA in human plasma (Figure 7). The analysis of plasma samples from the volunteers showed almost the same peaks for DHA as well as for artemether, similar to the peaks observed for the standard solutions or in spiked plasma. The concentration of the drug in each sample was quantified from the peak area of the samples. The data are shown in Table 1. Therefore, the proposed method using LTQ Orbitrap in a selective ion monitoring mode along with Xcalibur software accurately determined and quantified the concentration of DHA in human plasma. The presence of DHA was also confirmed by MS/MS and Xcalibur software. The main pharmacokinetic parameters for DHA were also calculated. The mean C_max_ of 0.84 μg/mL for DHA was reached in 2.5 h (T_max_) after administration of the drug. The mean values of AUC_0-t_ and AUC_0-inf_ were 4.761 and 5.631 μg h/L, respectively. The elimination half-life was 2.02 h.

**TABLE 1:** Average concentration of DHA in plasma after each time interval.

Time (h)	Peak area of DHA	Peak area of IS	Concentration (ug/mL)
0	0	6443258	0
0.25	281359	6331037	0.04444122
0.45	1184693	6331037	0.18712464
1	2461788	6331037	0.38884436
1.2	3666243	6331037	0.57909044
1.4	4478829	6331037	0.70744003
2	5086957	6331037	0.80349507
2.5	5383712	6331037	0.85036812
3	4254400	6331037	0.67199102
6	2281799	6331037	0.36041473
9	629242	6331037	0.09939004
12	502432	6331037	0.07936014

## 4 Discussion

Formulations of artemether are generally well tolerated with no significant adverse effects. However, there is a significant inter-individual variability in the pharmacokinetic parameters of artemether and DHA. According to the literature, the artemether is detected after *ca* 30 min in most cases and reaches the maximum plasma concentration of *ca* 400 ng/mL in approximately 2 h after which there is a decline in the plasma concentration.

An Orbitrap mass spectrometer analysis of DHA in plasma was successfully carried out, and it accurately determined small amounts of DHA in human plasma. The method is also adequately sensitive for the quantification of DHA in plasma. Different experimental procedures regarding the instrument and method were optimized. The method was also validated on the basis of specificity, accuracy and precision, calibration, recovery, ion suppression, and stability. Briefly, the method works well for the extraction of drugs, and the Orbitrap system with the help of Xcalibur software accurately and precisely determines the concentration of DHA in spiked as well as in volunteer’s plasma.

## 5 Conclusion

The LC-MS method reported in this paper accurately determined and quantified the metabolite of artemether and dihydroartemisinin and validated according to internationally accepted criteria. The ESI technique has been proven effective and sensitive in producing ions close to the protonated molecule with sufficient intensity to be monitored quantitatively, accurately, and selectively and confirmed using Xcalibur software. The reported method has the advantages of a simple liquid–liquid extraction procedure, a short run time of 10 min, and a small sample volume for analysis.

## Data Availability

The raw data supporting the conclusion of this article will be made available by the authors, without undue reservation.
